# Effectiveness of electroacupuncture as a treatment for osteoporosis: A systematic review and meta-analysis

**DOI:** 10.1097/MD.0000000000024259

**Published:** 2021-01-22

**Authors:** Linyan Fan, Zhifang Wu, Min Li, Ganghui Jiang

**Affiliations:** a Department of Preventive Medicine; b Department of Orthopedics and Trauma; c Department of Acupuncture and Massage, The Third Affiliated Hospital of Guangzhou University of Chinese Medicine (The Third Clinical Medical Institute Affiliated to Guangzhou University of Chinese Medicine); d Rehabilitation Centers, The First Affiliated Hospital of Guangzhou University of Chinese Medicine (The First Clinical Medical Institute Affiliated to Guangzhou University of Chinese Medicine), Guangzhou Guangdong, China.

**Keywords:** electroacupuncture, meta-analysis, osteoporosis, systematic review

## Abstract

**Background:**

Osteoporosis (OP) results in an increased risk of fragility fractures, representing a major public health problem. In preventing OP, complementary and alternative medicine, such as acupuncture, was recommended because of the low efficiency and side effects of medications. Recently, there is insufficient evidence on electroacupuncture as an effective therapy for OP management. Hence, we evaluated the effectiveness of electroacupuncture for OP treatment.

**Methods:**

We conducted a systematic review and meta-analysis of clinical studies on patients with OP. Five databases (PubMed, Embase, Cochrane Central Register of Controlled Trials, China National Knowledge Infrastructure, and Wanfang) were searched from the earliest publication date to March 12, 2020. Randomized controlled trials (RCTs) were included if electroacupuncture was applied as the sole treatment or as an adjunct to other treatments compared with medications in patients with OP. The measurement outcomes included serum aminoterminal propeptide of type I procollagen (PINP) and C-telopeptide of type I collagen (CTX) levels, bone mineral density (BMD) of lumbar, and visual analog scale scores for OP-related pain. Acupoints were extracted when available.

**Results:**

In total, 11 RCTs involving 731 participants were included for further meta-analysis. The meta-analysis showed that the use of electroacupuncture as a sole treatment or as an adjunct to other treatments could relieve OP-related pain compared with medications [mean difference (MD) =  −0.58, 95% confidence interval (CI); MD =  −0.97 to −0.19, *P* = .003, *I*^2^ = 88%; MD =  −1.47, 95% CI = –2.14 to −0.79, *P* < .001, *I*^2^ = 96%). Meanwhile, the results showed a favorable effect of electroacupuncture on decreasing serum beta-CTX levels. However, there were no significant differences in serum PINP levels and BMD of lumbar. Shenshu (BL23) was the most frequent acupoint stimulation among these studies.

**Conclusions:**

The application of electroacupuncture as an independent therapy or as an adjunct to other treatments might attenuate OP-related pain and serum beta-CTX levels. However, to overcome the methodological shortcomings of the existing evidence, due to a small size of samples and high risk of bias in these included RCTs, further rigorous studies are required.

## 1 Introduction

Osteoporosis (OP) is a condition characterized by low bone mass and structural deterioration of bone tissues, leading to an increased risk of fractures.^[1]^ OP is divided into primary and postmenopausal type. The incidence of OP is growing, affecting approximately 200 million people in China.^[2]^ OP can be noted at any age, but the prevalence is higher in adults, and it is noted more in women than in men.^[2]^ The most significant symptom of OP is pain in the bones, including back pain, hip pain, or other skeletal pain. Osteoporotic fractures due to bone fragility are the most severe complications of OP. Around 9 million patients with OP had osteoporotic fracture annually around the world, and > 300,000 patients with fractures required hospitalization in the UK each year.^[3,4]^ Osteoporotic fractures commonly occur in the spine, hip, elbow, and shoulder, causing pain and severe disability. According to Sernbo and Johnell,^[5]^ especially in the UK, the number of patients with an osteoporotic hip fracture will rise from 91,500 per year in 2015 to 101,000 per year in 2020, requiring hospitalization and operation.^[5]^ OP represents a major public health problem, affecting not only the people but also the economy of the society; thus, an effective and better therapy for OP prevention is needed. On the basis of the underlying mechanisms of OP, the medications used for OP treatment include estrogen, bisphosphonates, and calcium.^[6]^ However, because of the low efficiency and side effects of these medications, complementary and alternative medicine, such as Chinese herb, acupuncture, and moxibustion, is also recommended. Acupuncture, including needle acupuncture and warm needle acupuncture, is considered as an effective therapy in relieving pain and improving bone mineral density (BMD) of patients with OP. Moreover, electroacupuncture is another effective acupuncture commonly used in clinical practice for treating OP. Although there was a critical appraisal for warm needle acupuncture in OP management,^[7]^ electroacupuncture has no sufficient evidence as an effective therapy in OP management. Thus, we aimed to systematically review the literature to examine the effect of electroacupuncture as a treatment for OP.

## 2 Methods

### 2.1 Search strategy

Five databases (PubMed, Embase, Cochrane Central Register of Controlled Trials, China National Knowledge Infrastructure, and Wanfang) were searched from the earliest publication date to March 12, 2020, to find the relevant studies. Additionally, the reference list and reviews of the selected studies were also screened. The searching process was conducted independently by 2 authors, and after their discussion, the final searching list of the included studies was finalized. The third author resolved the disagreements, if any.

### 2.2 Study selection

#### 2.2.1 Inclusion criteria

The study was included if

1. it was a randomized controlled trial (RCT),
2. it discussed patients with OP (osteopenia or bone loss),
3. the intervention was either electroacupuncture therapy or electroacupuncture with other treatments, and
4. there was a comparison between only medications and other treatments without acupuncture.

#### 2.2.2 Exclusion criteria

The study was excluded if

1. it did not use RCT,
2. it did not discuss primary OP and postmenopausal OP,
3. the intervention group of studies combined 2 different forms of acupuncture,
4. the intervention group of studies combined > 2 therapies, and
5. the control group of studies included the same form of acupuncture in the intervention group.

### 2.3 Data extraction and outcome measures

The data of the included studies were independently extracted by the authors, which included author, sample size, age, intervention group, control group, and outcome measurements. Relevant acupoint data were extracted when available. The outcome measurements included biochemical markers of bone metabolism, such as beta C-telopeptide of type I collagen CTX (β-CTX), aminoterminal propeptide of type I procollagen (PINP), BMD, and visual analog scale (VAS) score for OP-related pain.

### 2.4 Assessment of bias risk and methodological quality of included studies

The methodological quality of the included studies was independently assessed by 2 authors according to the Cochrane Collaborations tool based on the following factors: random sequence generation, allocation concealment, blinding of participants and personnel, blinding of outcome assessment, incomplete outcome data, selective reporting, and other biases.^[8]^ The third author resolved the disagreements, if any.

### 2.5 Statistical analysis

The meta-analysis and statistical analysis were conducted using RevMan v5.2 (Cochrane, London, UK). Relative risks, 95% confidence interval (CI), were evaluated for dichotomous variables, and mean difference (MD), 95% CI, was used for continuous variables. Cochranes *I*^2^ statistic was used for assessing heterogeneity among the studies. If *I*^2^ was >50%, the random-effects model was applied; otherwise, the fixed-effects model was applied.

### 2.6 Ethics and dissemination

This is a literature-based study, ethical approval is not required.

## 3 Results

### 3.1 Study selection

**Figure 1:**
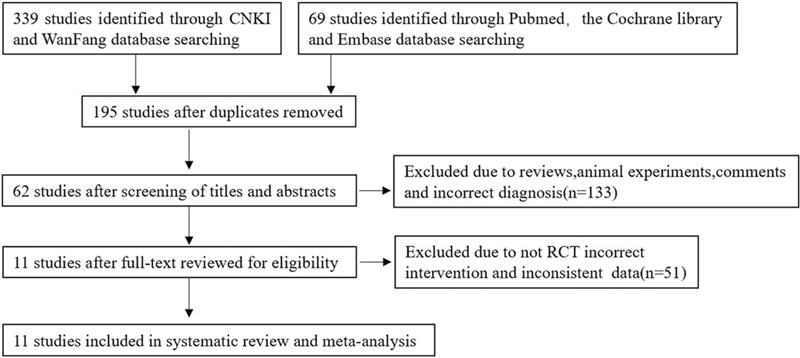
Flow diagram for the selection of the included studies through searching from the five databases (PubMed, Embase, Cochrane Central Register of Controlled Trials, China National Knowledge Infrastructure, and Wanfang). RCT = randomized controlled trial.

Figure 1 shows the flow diagram for the selection of the included studies. A total of 408 relevant studies were initially identified by searching the 5 databases. After removing the duplicates, the full text of 195 studies was reviewed. After screening the titles and abstracts, 133 studies were excluded because some did not have OP as diagnosis, some were experiments conducted on animals, and some were reviews and comment articles, yielding 11 final studies eligible for inclusion in the systematic review and meta-analysis (51 studies were excluded because they were not RCTs, did not meet the intervention criteria, and had inconsistent data.).^[9–19]^

### 3.2 Characteristics of included studies

Table 1 shows the 11 studies involving 731 participants and the characteristics of the studies, including author, age, intervention, outcomes, and acupoint data. Among these studies, 364 participants received electroacupuncture therapy and 167 were treated with electroacupuncture therapy combined with other treatments. Of the 11 studies, only 7 compared electroacupuncture therapy with medications. The VAS, BMD of lumbar, and serum PINP and CTX levels were used as outcomes in these studies.

**Table 1:** Characteristics of included studies.

First author (year)	Participants (n, age:mean ± SD)		Treatment group	Control group	Outcomes	Acupoints
Jin (2003)^[18]^	G1 (32, 67.7 ± 5.34)	G2 (30, 66.82 ± 6.51)	Electroaucpunctrure	Yiteng tablets and caltrate-D tablets	VAS, BMD of lumbar	EX-B2, Ah-shi, BL23, BL20, DU04, ST36, GB34
Cai (2007)^[9]^	G1 (34, 71.2 ± 4.7)	G2 (30, 70.8 ± 4.9)	Electroaucpunctrure	Calsinin and calcium carbonate, Vitamin D	VAS	BL40, EX-B2
Li (2012)^[16]^	G1 (32, −)	G2 (32, −)	Electroaucpunctrure+ Calcium carbonate D3 tablets	Calcium carbonate D3 tablets	BMD of lumbar	RN08, RN06, RN04, RN03, DU14, DU20, ST25, GB34, ST36, SP6
Zhou (2012)^[15]^	G1 (50, 58 ± 5)	G2 (50, 56 ± 7)	Electroaucpunctrure+calcitriol soft capsule	Calcitriol soft capsule	VAS, BMD of lumbar	BL20, BL21, BL23, BL18, DU03, DU04, BL40, Ah-shi, ST25, RN04, EX-CA1, ST36, SP6, KI3, GB39
Yang (2013)^[19]^	G1 (30, 61.32 ± 9.5)	G2 (30, 60.04 ± 9.98)	Electroaucpunctrure	Calcium carbonate D3 tablets and Qianggu capsule	VAS, BMD of lumbar	BL18, BL20, BL23, CV12, RN06, RN04
Cai (2014)^[12]^	G1 (30, 52 ± 7)	G2 (30, 50 ± 6)	Electroaucpunctrure	Calcium carbonate D3 tablets and Qianggu capsule	VAS, BMD of lumbar	BL11, BL23, GB39
Xiao (2014)^[17]^	G1 (20, -)	G1 (20,-)	Electroaucpunctrure	Alendronate Sodium Tablets+Vitamin D3	PINP	BL23, BL20, BL18, BL17, DU04, ST36, SP6, KI3, GB39
Zhang (2014)^[10]^	G1 (25, 62.2 ± 4.65)	G2 (25, 62.7 ± 5.07)	Electroaucpunctrure	Alfacalcidol capsules	BMD of lumbar, β-CTX	BL23, DU04, ST36, RN04, BL20, BL40, KI3
Zhao (2016)^[14]^	G1 (35, 60 ± 3)	G1 (35, 61 ± 3)	Electroaucpunctrure+Xianlingubao capsule	Xianlingubao capsule	PINP, β-CTX, BMD of lumbar	GB39, BL23, RN04
Wang (2016)^[11]^	G1 (30, 61.8 ± 4.71)	G2 (31, 62.2 ± 4.29)	Electroaucpunctrure	Calcium carbonate D3 tablets	VAS, BMD of lumbar	BL11, BL23, ST36
Li (2016)^[13]^	G1 (50, 72.06 ± 5.71)	G2 (50, 72.37 ± 4.62)	Electroaucpunctrure+Guyu decoction	Guyu decocton	VAS, BMD of lumbar	GB34, BL23, GB39

### 3.3 Risk of bias assessment

**Figure 2:**
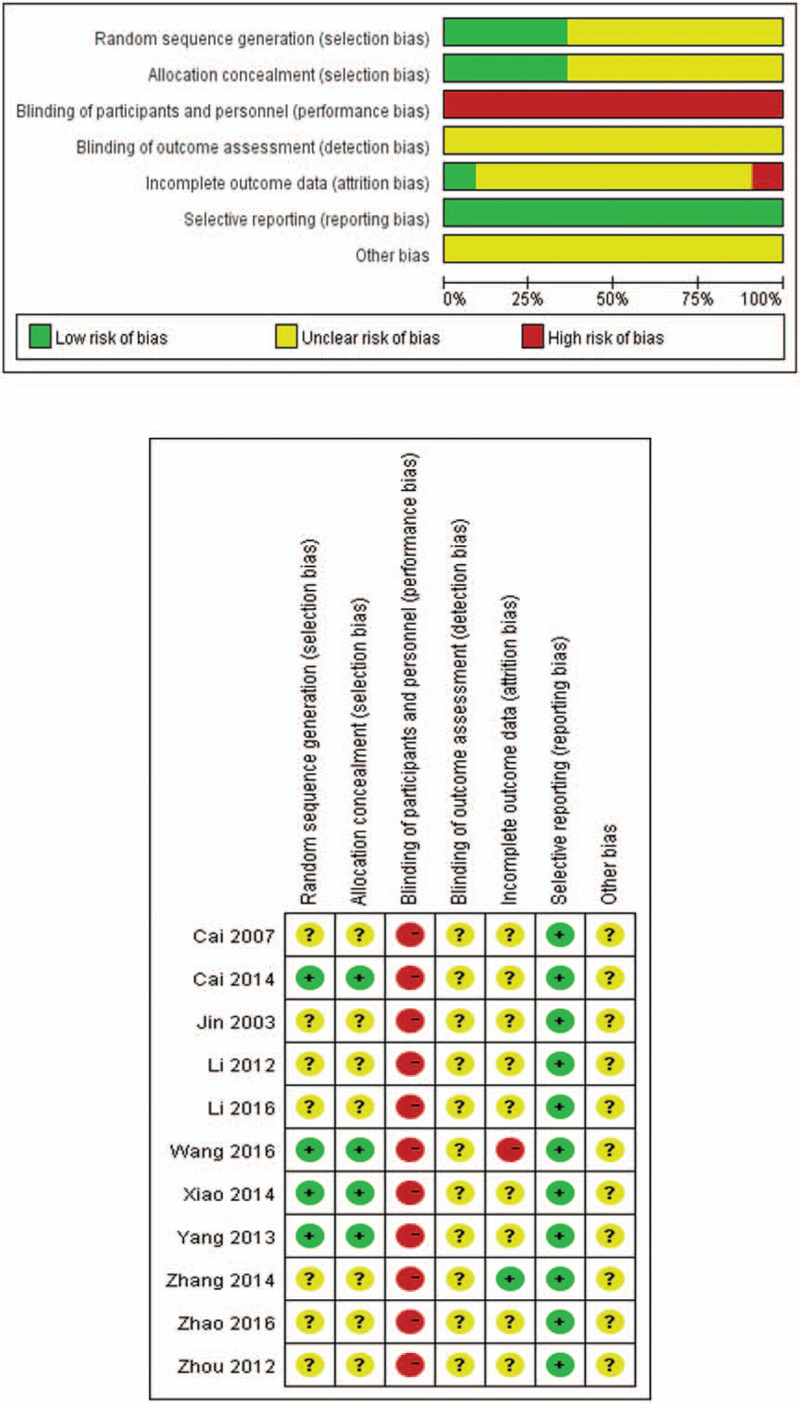
Risk of bias assessment.

According to the Cochrane Collaborations tool, the quality of the included studies was generally low. Only 4 of them reported random sequence generation and allocation concealment. Half of them had high risk in selection bias, performance bias, detection bias, and attrition bias due to lack of sham acupuncture in the control group and not mentioning random sequence generation and blinding of outcome assessment. One study mentioned the treatment method for dropout participants as well; however, 1 study excluded dropout participants from data analysis, which might have increased the risk of attrition bias (Fig. 2).

### 3.4 Back pain

**Figure 3:**
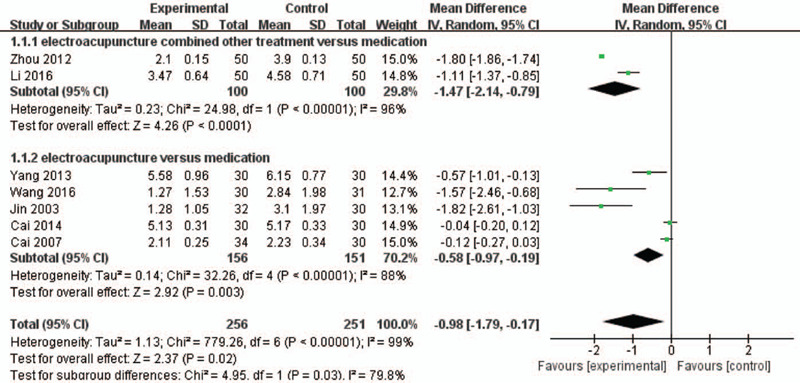
A meta-analysis of back pain between electroacupuncture and medications. CI = confidence interval; SD = standard deviation.

Seven articles were included to analyze the pain score. Figure 3 shows 2 studies that showed that electroacupuncture was effective as an adjunct to other treatments compared with medications based on VAS scores (MD = −1.47, 95% CI; MD = −2.14 to −0.79, *P *< .001, *I*^2^ = 96%). Meanwhile, a pooled results of 7 articles reported that only electroacupuncture therapy was more effective than medications based on back pain scores (MD = −0.58, 95% CI; MD = −0.97 to −0.19, *P *= .003, *I*^2^ = 88%).

### 3.5 BMD of lumbar

**Figure 4:**
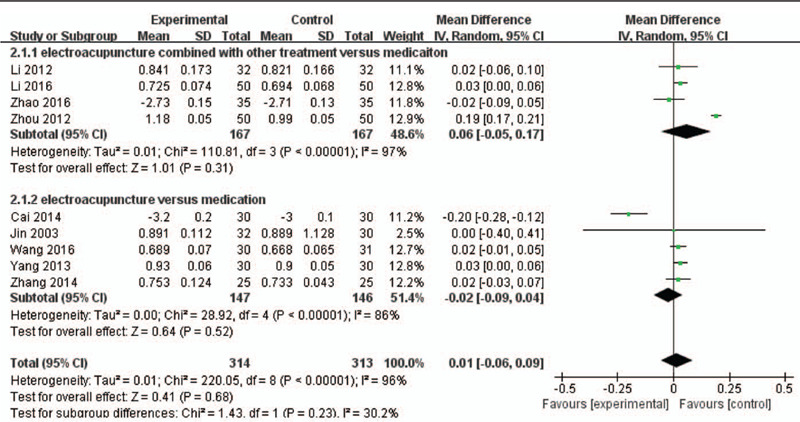
A meta-analysis of BMD of lumbar between electroacupuncture and medications. CI = confidence interval; SD = standard deviation.

Nine articles reported the BMD of lumbar as an outcome between electroacupuncture and medications (Fig. 4). The random-effects model was applied because of the high level of heterogeneity (*P *< .001, *I*^2^ = 97%; *P *< .001, *I*^2^ = 86%). The pool results of 4 RCTs yielded that there was no difference between electroacupuncture as an adjunct to other treatments compared with medications (MD = 0.06, 95% CI; MD = −0.05 to 0.17, *P* = .31, *I*^2^ = 97%). Also, there was no difference between electroacupuncture as an independent treatment and medications (MD = −0.02, 95% CI; MD = −0.09 to 0.04, *P* = .52, *I*^2^ = 86%).

### 3.6 Serum PINP levels

**Figure 5:**
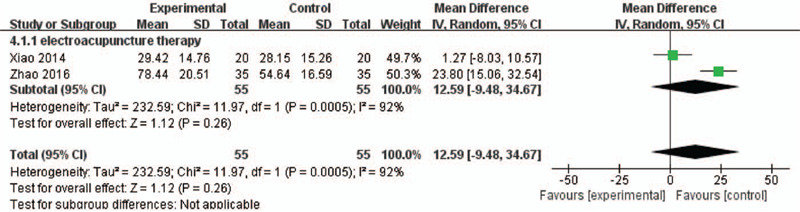
A meta-analysis of PINP between electroacupuncture and medications. CI = confidence interval; SD = standard deviation.

Figure 5 shows 2 trials that included serum PINP levels in meta-analysis. The random-effects model was employed for the parameters because of the high level of heterogeneity (*P* = .0005, *I*^2^ = 92%). Moreover, electroacupuncture therapy did not change the serum PINP levels compared with medications (MD = 12.59, 95% CI; MD = −9.48 to 34.67, *P* = .26, *I*^2^ = 92%).

### 3.7 Serum β-CTX levels

**Figure 6:**
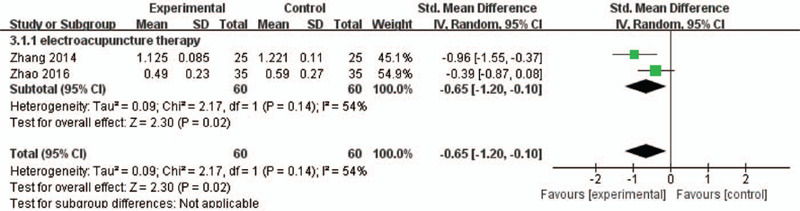
A meta-analysis of β-CTX between electroacupuncture and medications. CI = confidence interval; SD = standard deviation.

According to Figure 6, 2 RCT studies reported serum β-CTX levels treated with electroacupuncture. The random-effects model was used in this meta-analysis because of heterogeneity (*P* = .14, *I*^2^ = 54%). The serum β-CTX levels for electroacupuncture were lower than those for medications (MD = −0.65, 95% CI; MD = −1.20 to 0.10, *P* = .02, *I*^2^ = 54%).

### 3.8 Acupoints frequency

**Figure 7:**
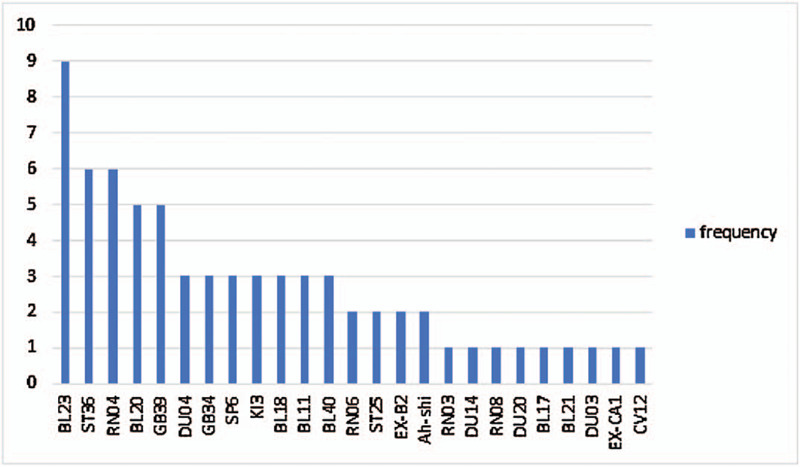
Frequency of acupoints of the included studies.

Figure 7 shows the frequency of acupoints adopted from the included studies. Shenshu (BL23) was the most frequent acupoint among the studies. Electroacupuncture for OP was applied at shenshu (BL23) in nine studies, at zusanli (ST36) and guanyuan (RN04) in 6 studies, and at pishu (BL20) and xuanzhong (GB39) in 5 studies.

## 4 Discussion

The objective of this systematic review and meta-analysis was to assess the effectiveness of electroacupuncture as a treatment for OP. OP is a major public health problem, and there is obvious evidence confirming that electroacupuncture could attenuate OP in animals.^[20,21]^ However, there are no convincing benefits of acupuncture in humans yet. We searched 5 databases (PubMed, Embase, Cochrane Central Register of Controlled Trials, China National Knowledge Infrastructure, and Wanfang) and obtained 11 studies that met the inclusion criteria (most of these studies were derived from the Chinese database).

In the present review, 11 studies involving 731 participants were identified. The VAS scores, BMD of lumbar, and serum PINP and β-CTX levels were used as outcomes for assessing the effectiveness of electroacupuncture for treating OP. As most of them were derived from the Chinese database, there was a high potential risk of publication bias. Moreover, almost all Chinese studies reported positive results, which might lead to more positive results in the meta-analysis. According to the Cochrane Collaborations tool, we found that the quality of the selected articles was low. Over half of them had risk in selection bias, performance bias, detection bias, and attrition bias. Thus, we defined the limitations found based on these studies. First, there were no details of random sequence generation and concealment, so we could not judge the method of RCT. Second, none of them had blinding performance, although there is currently no perfectively acknowledged method for sham acupuncture or achieving a placebo effect. Finally, all of the included RCTs used small sample sizes. Although it was a pool effect in the methodology, the pooled results demonstrated that electroacupuncture applied as the sole treatment or as an adjunct to other treatments could reduce the back pain scores in patients with OP. It was consistent with the fact that acupuncture is used as an analgesic therapy to relieve pain.^[22,23]^ However, compared with medications, there was no increase in the BMD of lumbar when electroacupuncture was used independently or in combination with other treatments. According to previous studies, PINP and CTX were recommended as bone formation and bone resorption markers, respectively, and the markers of bone turnover could predict OP and osteoporotic fractures.^[24]^ In our meta-analysis, we found that electroacupuncture attenuated the serum β-CTX levels in patients with OP, compared with medications, but it could not change the serum PINP levels. With increasing bone degradation, an elevated amount of β-CTX can be verified in the blood.^[25]^ As the most specific and sensitive bone resorption marker, serum β-CTX level has been proposed as an important marker of bone resorption for many years by the International Osteoporosis Foundation and International Federation of Clinical Chemistry.^[26]^ Electroacupuncture could decrease the serum β-CTX level, for example, Zeng et al^[27]^ reported that electroacupuncture as an adjuvant treatment with methotrexate plus leflunomide could reduce the levels of bone metabolisms markers, β-CTX and TRACP-5b, in the peripheral blood of patients with rheumatoid arthritis after 8 weeks, compared with medications. This may be the potential mechanism that suggests that electroacupuncture is an effective therapy for OP. However, further high-quality, larger-sample studies on electroacupuncture attenuating serum β-CTX levels in OP need to be performed. As bone resorption and formation are complicated processes, it is still unknown whether electroacupuncture as an adjuvant treatment or as an independent therapy improves BMD in patients with OP. However, other researches on animals have demonstrated the effects of electroacupuncture on osteopenia and OP through signaling pathways and gene expression regulation.^[28–30]^ Fan et al^[28]^ reported electroacupuncture stimulation at CV4, which activated the Wnt/β-catenin signaling pathway, increased serum ALP and BGP levels and BMD, affecting bone formation and promoting bone metabolism in rats with ovariectomy-induced OP. Furthermore, Zheng et al^[29]^ found that electroacupuncture stimulation at CV4/CV6 or BL23/BL20 increases levels of osteocalcin and the BMD of lumbar vertebrae, decreases levels of TRACP-5b, and improves bone microstructure in the femur in rats with ovariectomy-induced OP. The underlying mechanisms might be that it significantly upregulated β-catenin and Runx2 expression and downregulated phosphorylated (p)-p38 and p-JNK expression. Furthermore, Zhou et al^[30]^ demonstrated that electroacupuncture using the bilateral ST36 and SP6 acupuncture points can prevent OVX-induced bone loss and deterioration of bone architecture and strength by stimulating the Wnt/β-catenin signaling pathway. These findings suggest that electroacupuncture is a promising alternative therapy for OP in humans.

On the basis of the “kidney governing bones” theory in traditional Chinese medicine, OP is closely correlated to the liver, spleen, and kidney. Thus, its treatment principle for OP is to soothe the liver, tonify the spleen, and reinforce the kidney.^[31]^ From the included studies, most of the acupoints used belonged to the meridians associated with the liver, spleen, and kidney. Shenshu (BL23) in the taiyang bladder channel of the foot was the most frequent stimulation, and it activated the original qi of the kidney, thus nourishing the kidney. Zusanli (ST36) in the yangming stomach channel of the foot regulates the spleen and kidney, invigarating qi and generating blood. Given the above points, acupoints stimulated by electroacupuncture in the included studies offer benefits to patients with OP, soothing the liver, tonifying the spleen, and reinforcing the kidney.

In summary, electroacupuncture as an independent therapy or as an adjunct treatment may attenuate back pain in patients with OP. Although it could decrease serum β-CTX levels, which is a bone resorption marker, there was no difference in BMD of lumbar between electroacupuncture and medications. The level of bias was high; thus, we cannot make a definite conclusion yet. Further rigorous studies are required.

## Acknowledgments

We would like to thank Professor Ganghui Jiang (Guangzhou University of Chinese Medicine, Guangdong, China) for his guidance in this study.

## Author contributions

**Data Management:** Linyan Fan, Zhifang Wu.

**Design and concept:** Linyan Fan.

**Formal analysis and interpretation:** Linyan Fan, Min Li.

**Literature searching:** Linyan Fan, Zhifang Wu.

**Methodology:** Linyan Fan, Min Li.

**Writing – original draft:** Linyan Fan.

**Writing – review & editing:** Ganghui Jiang.
